# Kinetic Study of Yellow Fever 17DD Viral Infection in *Gallus gallus domesticus* Embryos

**DOI:** 10.1371/journal.pone.0155041

**Published:** 2016-05-09

**Authors:** Pedro Paulo de Abreu Manso, Bárbara Cristina E. P. Dias de Oliveira, Patrícia Carvalho de Sequeira, Yuli Rodrigues Maia de Souza, Jessica Maria dos Santos Ferro, Igor José da Silva, Luzia Fátima Gonçalves Caputo, Priscila Tavares Guedes, Alexandre Araujo Cunha dos Santos, Marcos da Silva Freire, Myrna Cristina Bonaldo, Marcelo Pelajo Machado

**Affiliations:** 1 Laboratório de Patologia, Instituto Oswaldo Cruz, FIOCRUZ, Rio de Janeiro, Brazil; 2 Laboratório de Biologia Molecular de Flavivírus, Instituto Oswaldo Cruz, FIOCRUZ, Rio de Janeiro, Brazil; 3 Universidade Federal do Estado do Rio de Janeiro, UNIRIO, Rio de Janeiro, Brazil; 4 Laboratório de Tecnologia Virológica, Instituto de Tecnologia em Imunobiológicos, Fundação Oswaldo Cruz, Rio de Janeiro, Brazil; University of Texas Medical Branch, UNITED STATES

## Abstract

Yellow fever continues to be an important epidemiological problem in Africa and South America even though the disease can be controlled by vaccination. The vaccine has been produced since 1937 and is based on YFV 17DD chicken embryo infection. However, little is known about the histopathological background of virus infection and replication in this model. Here we show by morphological and molecular methods (brightfield and confocal microscopies, immunofluorescence, nested-PCR and sequencing) the kinetics of YFV 17DD infection in chicken embryos with 9 days of development, encompassing 24 to 96 hours post infection. Our principal findings indicate that the main cells involved in virus production are myoblasts with a mesenchymal shape, which also are the first cells to express virus proteins in *Gallus gallus* embryos at 48 hours after infection. At 72 hours post infection, we observed an increase of infected cells in embryos. Many sites are thus affected in the infection sequence, especially the skeletal muscle. We were also able to confirm an increase of nervous system infection at 96 hours post infection. Our data contribute to the comprehension of the pathogenesis of YF 17DD virus infection in *Gallus gallus* embryos.

## Introduction

The yellow fever virus (YFV) is the etiological agent of yellow fever, one of the most lethal diseases in tropical regions of the world in the last century. This viral infection causes a pansystemic febrile disease with hepatic, renal and myocardial injuries. In severe cases, it can cause hemorrhage and shock, involving a mortality rate of about 50%. There is no antiviral treatment for this disease, so the only control is preventive action based on vaccination of populations living in risk areas [1–3].

The yellow fever (YF) vaccine was formulated from the virulent Asibi yellow fever virus strain, named after the patient from whom it was isolated in Ghana in 1927. The 17D vaccine strain was obtained through serial passages of the Asibi strain in chicken tissue cultures and became attenuated for humans. Two important sub-strains were independently derived from the YFV 17D strain, 17DD and 17D-204. The YFV 17D strain was first adopted for vaccination in Brazil in 1937. Since then, the yellow fever 17DD vaccine has been produced by the Oswaldo Cruz Foundation. The vaccine is produced through inoculation of the yellow fever virus (17DD sample) into embryonated chicken eggs, free of specific pathogens (SPF), according to the World Health Organization standards [4–6].

One of the most important contributions to a better understanding of virus production in chicken eggs is the publication by Fox and Laemmert [7]. Their group addressed important aspects of yellow fever vaccine production in chicken embryos, such as the best virus strain, embryological stage for infection and time of infection. Although chicken embryos have been used since 1937 as a source of yellow fever virus, the histopathological and molecular bases that regulate viral infection in this biological system are still not well understood.

Recently, our group [8] demonstrated by molecular techniques and immunofluorescence assays that the YFV 17DD replicates mainly in skeletal muscle tissue, and also in the nervous system, fibroblast cells and cardiomyocytes of White Legorn SPF chicken embryos at 72 hours post infection, representing a similar condition employed in yellow fever vaccine manufacture. It was possible to observe for the first time the histopathological alterations in this model and to determinate that muscle tissue could be the major site of virus replication. However, in order to deepen the understanding of the YFV 17DD spread pattern and replication in different embryo tissues, the same approach was applied at other time points of infection. Besides the biological importance, knowledge of which cells and tissues are involved in YFV 17DD replication can provide new insight to develop different strategies for vaccine production with less chicken proteins.

The aim of this study was to elucidate the kinetics of YFV 17DD proliferation in *Gallus gallus domesticus* embryos, from 24 to 96 hours post infection. We ascertained that myoblast cells with a mesenchymal shape appeared to be the first infected cells in *Gallus gallus* embryos 48 hours after infection. At 72 hours, we observed an increase of infected tissues. Numerous cells are affected during the infection, mainly muscular skeletal cells. We also observed a numerical increase of infection in nervous system cells after 96 hours. Our data contribute to comprehension of the kinetics of YFV 17DD infection in *Gallus gallus* embryos.

## Materials and Methods

### Biological System

Specific pathogen-free (SPF) fertilized White Leghorn chicken eggs (*Gallus gallus domesticus*; Linnaeus, 1758) were obtained from the YF vaccine production unit (Fiocruz). There, eggs were infected in the yolk sac with 17DD EPlow virus seed lot (1–5 x 10^3^ PFU per inoculum) on the ninth day of development [4, 8–10]. Eggs were kept in an IP70 brooder (Premium Ecologica, Brazil) with controlled temperature at 37.5°C, and 55% relative humidity. As negative controls, embryos kept under the same conditions were inoculated with water by injection. All embryos were euthanized by cutting their umbilical vessels and their amniotic sacs followed by immediate decapitation. For all experiments, embryos and viteline and chorioallantoic membranes were collected at 24, 48, 72 and 96 hours post infection (10, 11, 12 and 13 days of development respectively). Each point of infection was composed of seven infected embryos and the same number of controls.

### Ethics Statement

This work was conducted with fertilized White Leghorn chicken eggs (*Gallus gallus domesticus*) with 9 to 13 days of development, obtained already infected from Instituto de Tecnologia em Imunobiológicos (Fiocruz, Rio de Janeiro, Brazil). All experiments were in accordance with the yellow fever vaccine production protocol, which has been applied since 1937, when the vaccine production started at the mentioned unit, under ethical approval of Fiocruz. In addition, we used an animal euthanasia protocol described and approved by our Institutional Animal Care and Use Committee and in accordance with Law 11,794/08, which covers the scientific use of laboratory animals, including the principles of the Brazilian Society Laboratory Animal Science (SBCAL).

### Histopathological Analysis

The histopathological analysis was performed under a brightfield microscope with embryo trunks cleaved transversely in 3 mm sequential sections and separated from the head, wings and legs. Membranes were cleaved in regions defined by quadrants. All samples were fixed in Carson`s formalin-Millonig for 48 hours [11] and processed according to standard histological techniques for paraffin embedding. At least three sections (5 μm thick) from each block were stained with hematoxylin-eosin [12] and analyzed under an Axiovert Z1 microscope (Carl Zeiss, Germany) equipped with an mRC5 Axiocam digital camera (Carl Zeiss, Germany).

### Immunofluorescence Assay

Sections of all paraffin blocks from three infected animals and three control embryo specimens per point of infection were submitted to immunofluorescence assay, as previously described [8], with a mouse polyclonal anti-yellow fever virus antibody (Evandro Chagas Institute). Double staining was performed in some samples with an anti-desmin antibody (cat. RB-9014, Thermo Scientific, USA). As secondary antibody, AlexaFluor 488-conjugated goat anti-mouse (cat. A11001, Life Technologies, USA) or AlexaFluor 546-conjugated goat anti-rabbit (cat. A11010, Life Technologies, USA) was applied at 37°C for 1 h, followed by counterstaining with 1:5,000 DAPI (cat. 03571, Molecular Probes, USA). Negative controls were processed by omitting treatment with the primary antibodies. All sections were analyzed under an LSM 710 confocal microscope (Carl Zeiss, Germany).

### Nested-PCR

RNA samples were extracted from formalin-fixed, paraffin-embedded tissue (FFPE). RNA was extracted from the same blocks in the immunofluorescence analysis, which were either positive or negative to the YF 17DD virus, as previously described [8]. Samples eluted after the procedure were amplified by reverse transcription-PCR (Thermoscript RT-PCR kit—cat. 11146016, Life Technologies, USA) with universal Flavivirus Yellow Fever primers (YF1–5`GGTCTCCTCTAACCTCTAG 3`and YF3- 5`GAGTGGATGACCACGGAAGACATGC 3`) [13]. Afterwards, a second amplification was carried out with yellow fever internal primers previously designed by our group [8] (YF2–5`CGAGTTTTGCCACTGCTAAGCT 3`and YF4–5`TAGACCCCGTCTTTCTACCACC 3`). Two different protocols were adopted with specific primers in RT-PCR using forward and reverse YF-1 and YF-3 primers to detect the genomic and replicative intermediate RNA. All samples were sequenced after nested-PCR amplification in an ABI 3730 DNA analyzer (Applied Biosystems—USA). The sequences were aligned to the Yellow Fever Virus 17DD genomic sequence (GenBank U17066.1) incorporating ClustalW2 [14].

## Results

Overall, the YFV 17DD infection in *Gallus gallus* embryos was mild and few lesions could be identified in tissues analyzed by brightfield microscopy. Some of these alterations were at times not easy to distinguish from normal development events. The discrimination between these two aspects was only possible through molecular and immunological techniques, which provide complementary data. Intriguingly, we could not detect viral protein by immunofluorescence in the liver, yolk sac and chorioallantoic membrane in any of the animals studied. Membranes were also negative when submitted to nested-PCR assay. The analysis of infected embryos at different times of infection exhibited interesting aspects of virus particle production and distribution in many embryonic tissues, with a temporal dispersion association.

After 24 hours of infection, neither histopathological changes nor viral antigen presence were observed. Likewise, after extraction in paraffin embedded material, we could not detect either viral genome or replicative intermediate RNA.

**Fig 1:**
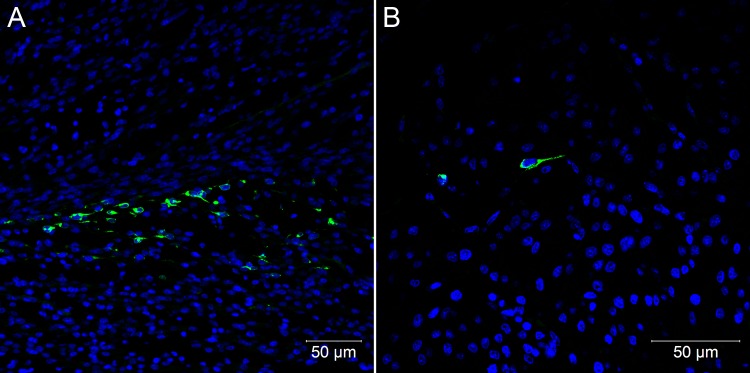
Confocal microscopy analysis of embryos of *Gallus gallus domesticus* 48 hpi with Yellow Fever 17DD virus. Mesenchymal cells in leg skeletal muscle (A) and in heart (B). Yellow fever virus in green, nuclei stained with DAPI in blue.

Histopathological changes were not evident 48 hours after embryo infection, although at this time point, viral antigen was detected by immunofluorescence in the first positive cells, which were characterized by few mesenchymal-like cells in the skeletal muscle of the legs (Fig 1A) and in the heart (Fig 1B). However, RNA extracted from paraffin-embedded samples of immunofluorescent positive blocks was negative for the presence of viral genomic and replicative intermediate RNA.

**Fig 2:**
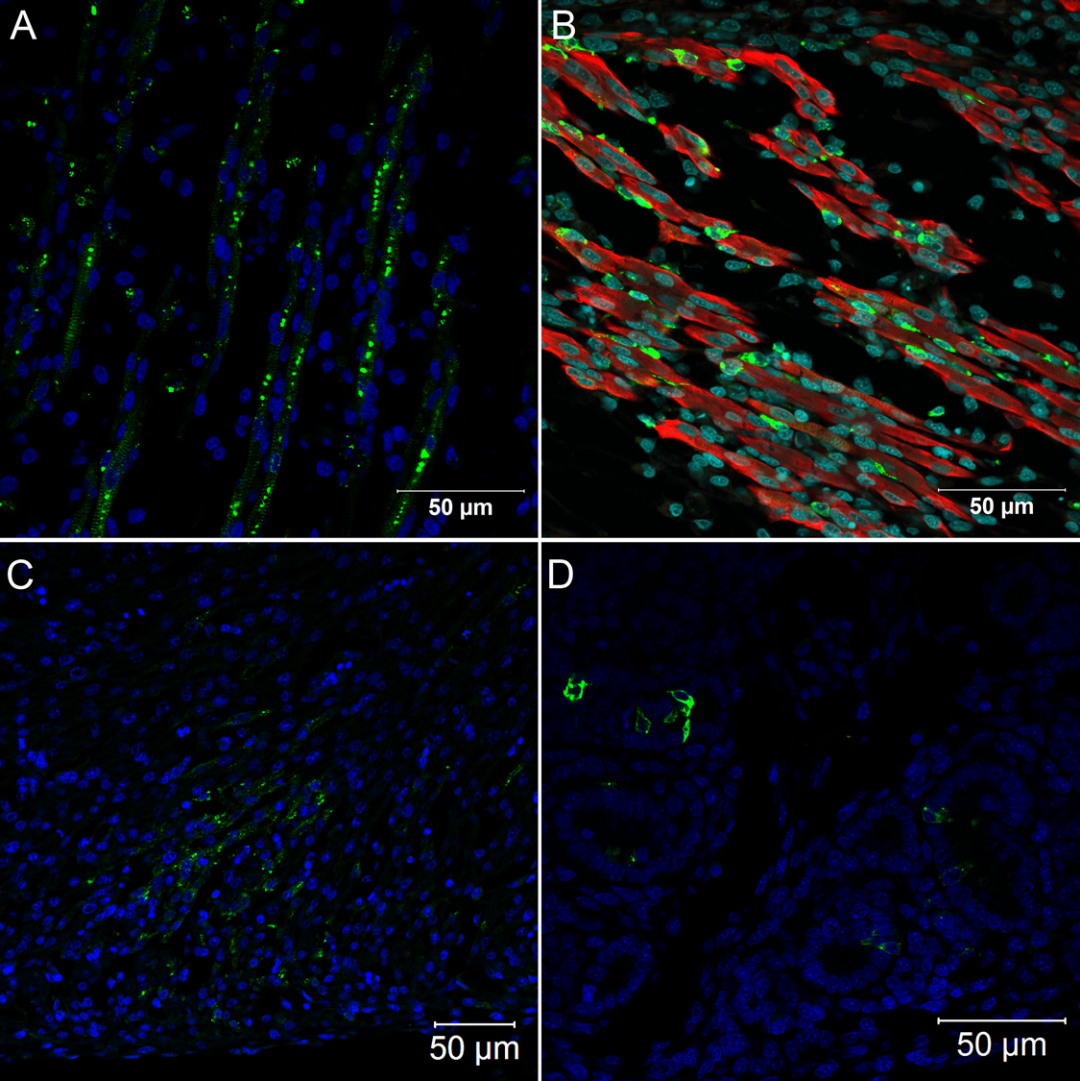
Confocal microscopy analysis of *Gallus gallus domesticus* 72 hpi with Yellow Fever 17DD virus. Infected skeletal muscle bundles (A). The viral protein form clusters in the cytoplasm and follow the muscular striations; Infected muscular fibers and mesenchymal cells adhering to infected and uninfected muscular fibers (B); Infected muscular cells in the heart (C). Infected kidney tubular epithelium cells (D). Yellow fever virus proteins in green, nuclei stained with DAPI in blue and desmin in red.

**Fig 3:**
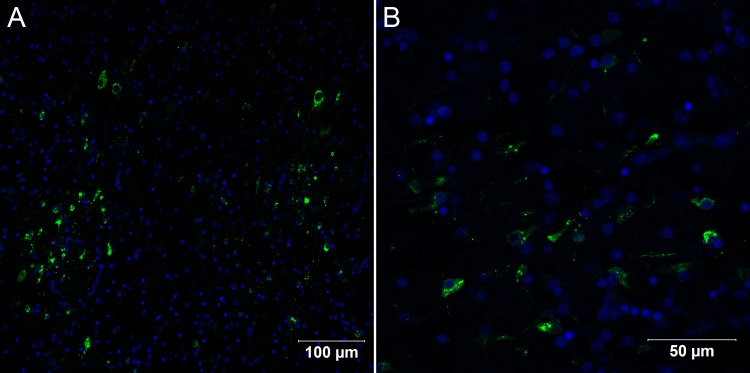
Confocal microscopy analysis of brain in *Gallus gallus domesticus* 72 hpi with Yellow Fever 17DD virus. Infected nervous tissue cells in the brain (A); Details of infected neurons in the brain (B). Yellow fever virus proteins in green, nuclei stained with DAPI in blue.

**Fig 4:**
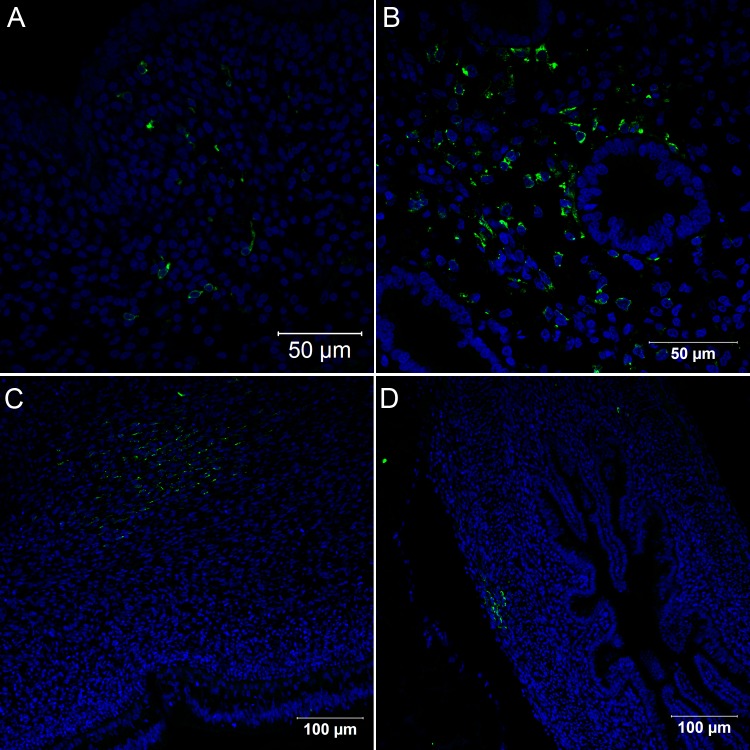
Confocal microscopy analysis of different tissues in *Gallus gallus domesticus* 72 hpi with Yellow Fever 17DD virus. Infected cells in dermal connective tissue (A); Infected lung parenchyma cells (B); Infected cells in the muscular layer of the gizzard (C); Infected fibroblastoid cells in the yolk stalk (D). Yellow fever virus proteins in green, nuclei stained with DAPI in blue.

Between 48 and 72 hours post infection, there was a marked proliferation of virus particles, observed by immunofluorescence microscopy, revealing the emergence of various infected organs. Hence, at 72 hours of infection, it was possible to detect viral antigen in skeletal muscle (Fig 2A and 2B), cardiac muscle (Fig 2C), renal tubular epithelium (Fig 2D), central nervous system cells (Fig 3A and 3B), connective tissue fibroblastoid cells (Fig 4A), lung parenchyma mesenchymal cells (Fig 4B), the gizzard (Fig 4C) and the yolk stalk (Fig 4D). These data suggest that the skeletal muscle is the main site of virus replication since muscle tissue of different sites displayed entirely infected bundles. At this point of infection, it was possible to visualize infected mesenchymal-like cells adhering to the muscular fibers and infected mature muscular fibers (Fig 2B). Hearts presented small clusters of positive cells spread throughout the organ. The nervous system presented infection in the brain, cerebellum, meninges and spinal cord neurons. Renal tubular epithelium cells were strongly positive and could be seen in small clusters or isolated in the tubular epithelium. Infected fibroblastoid cells were scattered, present in many tissues such as the perichondrium and dermis.

**Fig 5:**
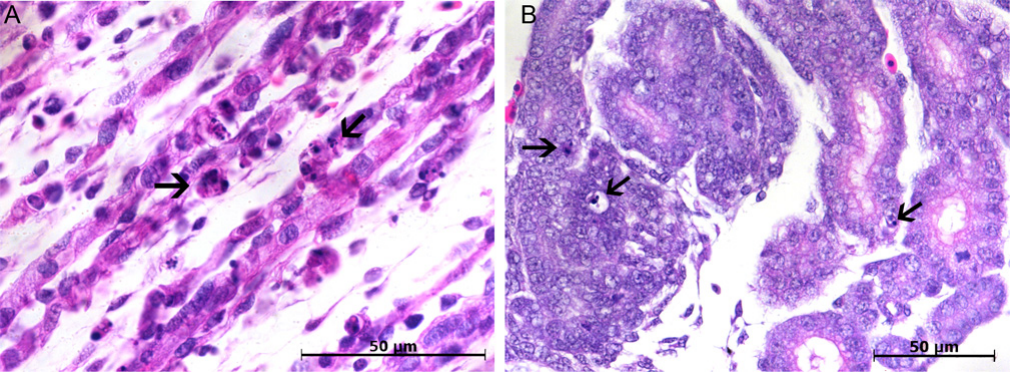
Brightfield microscopy analysis of *Gallus gallus domesticus* 72hpi with Yellow Fever 17DD virus. Apoptotic corpuscles in muscle bundles (A), and in the kidney tubular epithelium (B). Apoptotic nuclei are indicated by black arrow (→). Hematoxylin and eosin stain.

**Fig 6:**
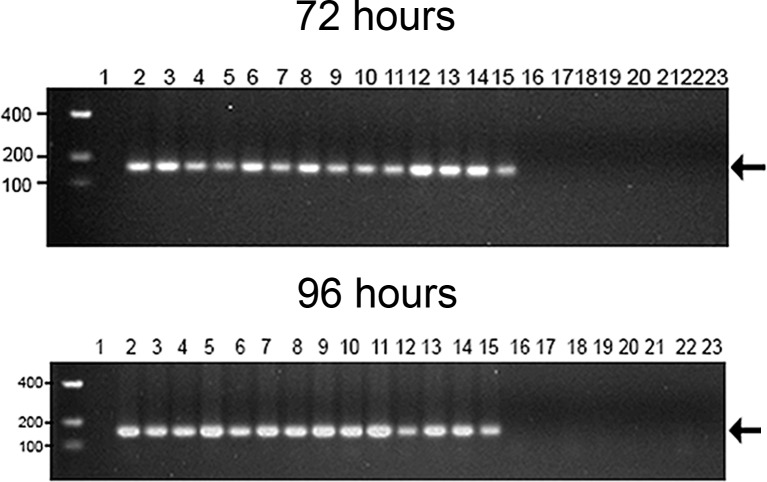
Detection of YF 17DD viral RNA by nested-PCR analysis. The RNA was extracted from formalin-fixed paraffin-embedded tissues. YF 17DD amplicons were assayed by means of agarose gel electrophoresis. The above figure show samples from a chicken embryo infected for 72 hours, and the bottom figure 96 hours. The lanes correspond to the following specimens: (1) empty; (2) and (3) head; (4) and (5) legs; (6) and (7) wings; lanes from (8) to (15) trunks; (16) and (17) vitelline membrane; (18) and (19) chorioallantoic membrane; from (20) to (23) negative control (water-inoculated animals). Even-numbered lanes indicate samples submitted to amplification of genomic RNA whereas odd-numbered lanes indicate samples submitted to amplification of the replicative intermediate RNA. The molecular length markers are indicated on the left of the figure. The black arrow indicates the 156bp amplicon obtained from the amplification of YF 17DD RNA.

The first pathological alterations, characterized by rare apoptotic bodies, were visible at this point of infection in infected tissues, such as myocytes (Fig 5A) and some renal tubule cells (Fig 5B). These apoptotic bodies were not accompanied either by inflammation or other cellular immunological reactions. RT-PCR analysis in paraffin-embedded tissues indicated that all immunofluorescence positive blocks were also positive for the detection of viral genome and replicative intermediate RNA (Fig 6).

**Fig 7:**
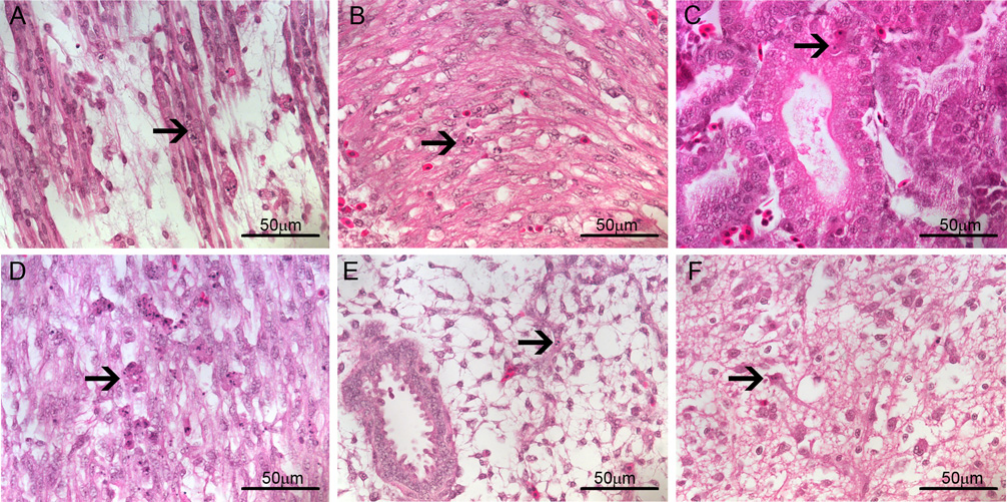
Brightfield microscopy analysis of *Gallus gallus domesticus* 96hpi with Yellow Fever 17DD virus. Apoptotic bodies in (A) muscular bundles; (B) heart cells; (C) kidney tubular epithelium; (D) gizzard muscle; (E) lung parenchyma; and (F) brain cells. Apoptotic nuclei are indicated by black arrow (→). Hematoxylin and eosin stain.

After 96 hours of infection, morphological alterations remained rare even though more evident than at 72 hours. Apoptotic bodies were still evident in a few myocytes (Fig 7A), cardiomyocytes (Fig 7B), renal tubules (Fig 7C), gizzard parenchyma (Fig 7D), lung (Fig 7E) and brain (Fig 7F). At this point of infection, a granulocytic accumulation was found in an isolated area of cardiac muscular tissue of one animal.

**Fig 8:**
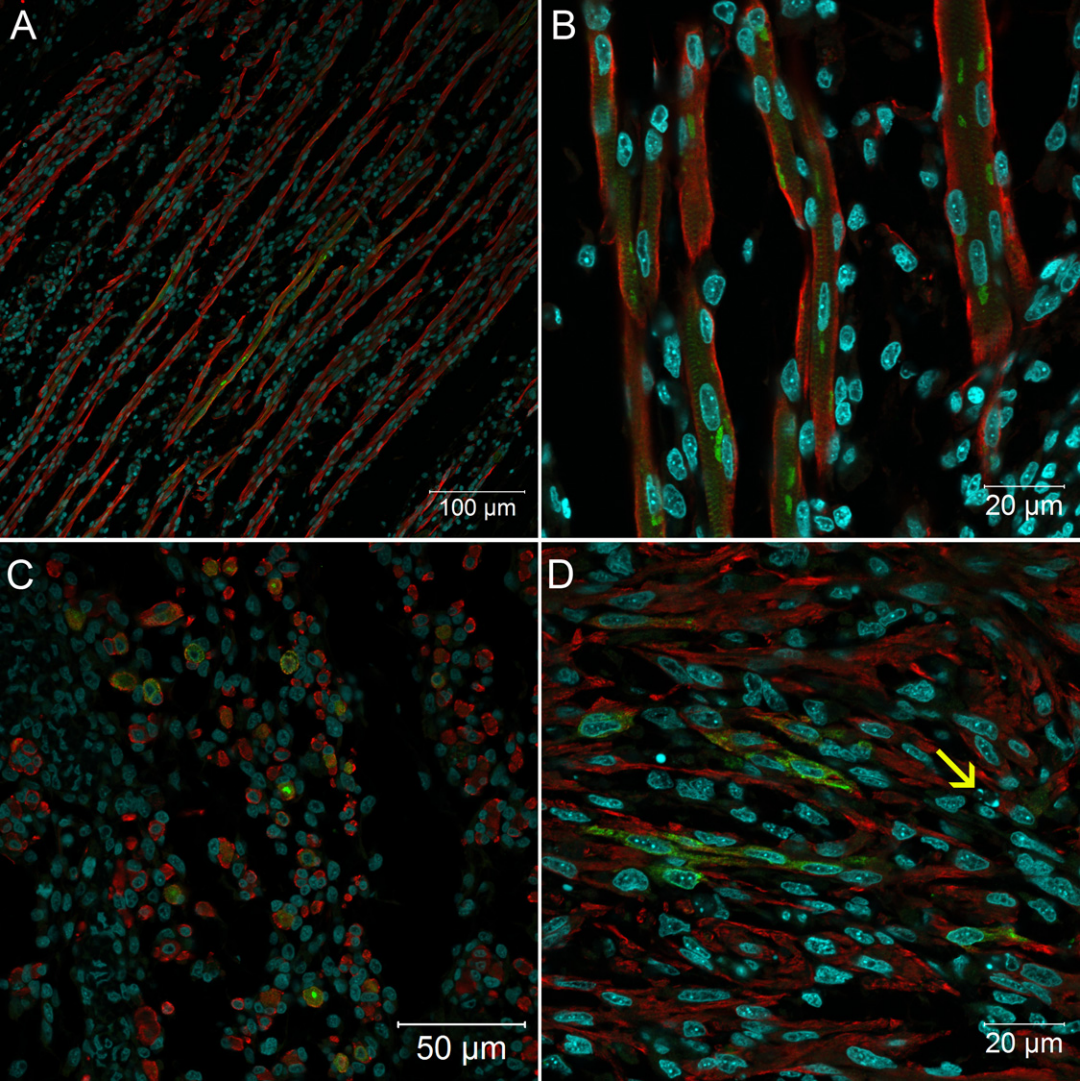
Confocal microscopy analysis of muscular tissue in *Gallus gallus domesticus* 96hpi with Yellow Fever 17DD virus. Infected skeletal muscle bundles, shown viral protein aggregation (A); detail of infected muscular fibers shown perinuclear immunostaining of viral protein and clusters follow the muscular striations, longitudinal section (B) and transversal section (C); infected cardiomyocytes in the heart (D). Apoptotic bodies closer to infected cells (→).Yellow fever virus proteins in green, nuclei stained with DAPI in blue and desmin in red.

**Fig 9:**
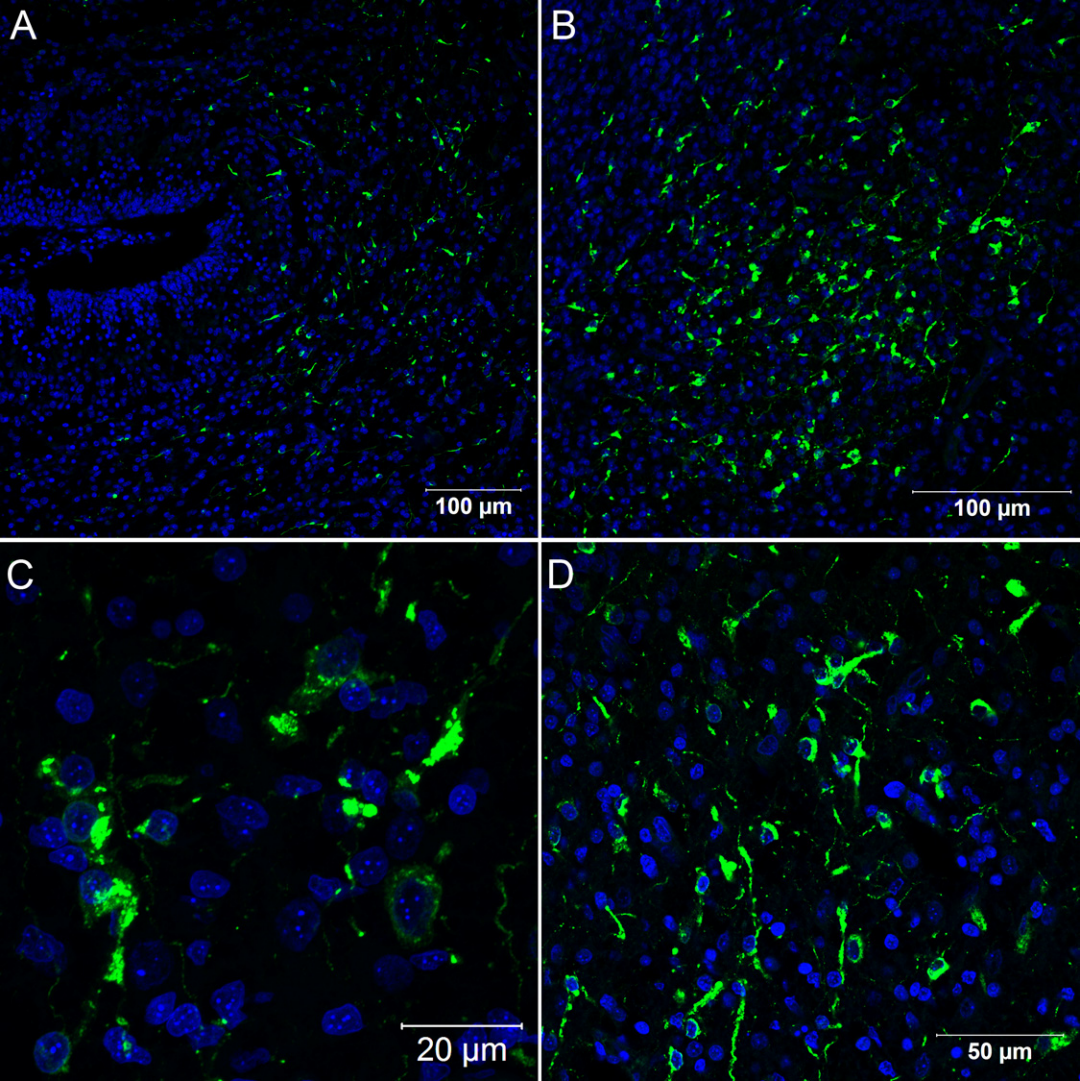
Confocal microscopy analysis of brain in *Gallus gallus domesticus* 96 hpi with Yellow Fever 17DD virus. Infected nervous tissue cells in the brain (A, B); Detail of infected cells in the brain congested with virus proteins (C,D). Yellow fever virus proteins in green, nuclei stained with DAPI in blue.

**Fig 10:**
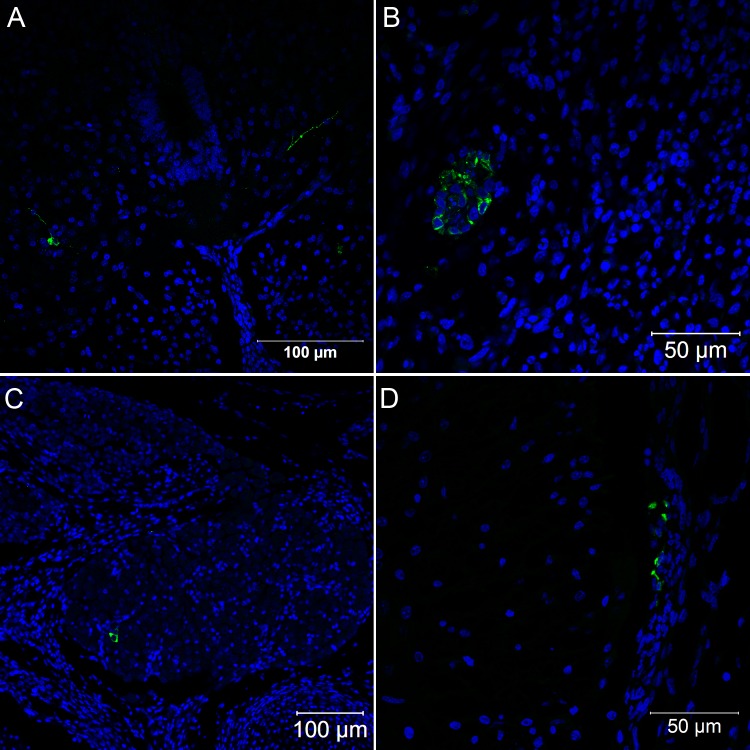
Confocal microscopy analysis of nervous system in *Gallus gallus domesticus* 96 hpi with Yellow Fever 17DD virus. Infected neurons in the spinal cord (A); infected cells in nerve bundles (B); infected cells in the dorsal root ganglion (C); infected fibroblastoid cells in the meninges (D). Yellow fever virus proteins in green, nuclei stained with DAPI in blue.

When analyzed by immunofluorescence assay, all organs and tissues positive at 72 hours continued to express viral antigen at 96 hours. The muscular bundles were thicker and remained infected (Fig 8A) with perinuclear clusters of viral protein (Fig 8B and 8C). The viral protein staining continued to follow the striations of the cytoskeleton, suggesting accumulation in the sarcolemma (Fig 8B). In the heart, a few apoptotic cells were visualized closer to infected cardiomyocytes (Fig 8D). At this point, an exacerbation of infection in the nervous system was evident, with an increase in the number of brain cells expressing viral proteins (Fig 9). Likewise, infected spinal cord neurons were more numerous at 96 hours than at 72 hours (Fig 10A). Cells of some nerve bundles (Fig 10B), dorsal root ganglion (Fig 10C) and meninges (Fig 10D) were also positive at this interval of infection. Samples of paraffin-embedded tissues submitted to molecular analysis confirmed the presence of viral genome and replicative intermediate in all blocks positive for viral antigen by immunofluorescence microscopy (Fig 6).

## Discussion

This manuscript furnishes unprecedented data on the kinetics of the histopathological changes that occur in chicken embryos infected with YF 17DD virus during vaccine production. This infection in general proved to be mild and systemic. Our data indicate that the first cells to express infection are myoblasts with mesenchymal shape. These cells were observed in the heart and skeletal muscles, at 48 hours of infection. After 72 hours, cardiomyocytes, muscular fibers, renal tubule cells, parenchymal lung cells, nervous system cells, gizzard muscle cells, dermal fibroblasts and other cells were infected. After 96 hours, the infection became more intense in the nervous system, with continuing infection in the other tissues.

Fox and Laemmert [7], did not find histopathological changes by light microscopy in infected embryos, and in our study histopathological changes were also not so evident, often confused with normal developmental events. Hence, the viral detection in infected cells and tissues was only possible by employing molecular techniques.

In the *Gallus gallus* embryo model, the virus is inoculated in the yolk sac. However, it was not possible to identify viral proteins by immunofluorescence techniques in cells of the vitelline membrane, the endothelial wall or inside the blood vessels at all studied infection intervals. Viral genome was not detected by nested-PCR in the vitelline membrane at any studied time. Therefore, it is interesting to reflect on how viruses reach the organs that are infected at the subsequent time points. The vitelline membrane is highly vascularized, and at this stage of development it is responsible for much of the circulating blood cell production [15]. Taking into account the absence of infection in these cells, we hypothesize that once in the membrane, the inoculum is passively transported by the circulation to reach competent organs for viral production. In this context, after 24 hours neither viral RNA nor viral protein could be detected by nested-PCR or immunofluorescence.

Similarly, we did not observe any histopathological changes in different samples at 24 hours of infection. We hypothesize that the number of infected cells was not sufficient to be detected by nested-PCR in formalin-fixed paraffin-embedded (FFPE) samples or by immunofluorescence. Nevertheless, Fox and Laemmert observed a neuropathic effect on mouse brains by injecting body extract from 24 hour YF 17D-204 strain infected chicken embryos [7].

From 48 hours of infection onwards, infected cells were identified in skeletal and heart muscles, exhibiting a consistent pattern with myoblasts having a mesenchymal shape. In regions where the viral antigens were identified, few cells were positive. At this point of infection, immunofluorescence was more sensitive than nested-PCR for virus detection in FFPE samples.

In chicken embryos infected with the YF 17D-204 strain, there is an increase of viremia between 48 and 72 hours post-infection [7]. We observed the same occurrence in YF17DD-infected cells and tissues over this period. After 72 hours of infection, the viral antigen was also detected by immunofluorescence in renal tubules, the gizzard muscle layer, lung parenchyma and fibroblastoid cells in the dermis connective tissue. The presence of apoptotic bodies was observed in the same place where cells were positive to the viral protein by immunofluorescence, similar to that in humans and hamsters infected with wild yellow fever virus, where the predominant mechanism of cell death is intrinsic and extrinsic apoptosis [16–19]. Although molecular markers of apoptosis were not employed in our study, the lack of immune system maturity in this development stage of *Gallus gallus*, as well as the absence of the cellular reaction in infected regions, allow us to assume that the apoptosis trigger in this case is intrinsic. In general, the presence of apoptotic bodies in humans, other primates and hamsters is accompanied by microesteatosis of affected organs [16, 19, 20], which was not evident in our study model.

Both genomic and replicative intermediate RNA strands were detected after 72h of infection in all specimens that were positive for viral antigen by immunofluorescence. The presence of the replicative intermediate, in these samples, confirmed viral replication in the referred tissues. This result was confirmed by sequencing all amplicons presenting 100% identity with the yellow fever virus 17DD genome.

After 96 hours of infection, the cytopathic effect was rare, but the presence of apoptotic bodies was apparent in some cells. When compared to the YF 17D-204 growth kinetics in chicken embryos, at 96 hours of infection the virus titer was stabilized and there were higher concentrations of titer in the head and muscles of animals [7]. Likewise, we found a large increase in the number of infected cells in the brains of the animals studied.

During production of the yellow fever vaccine, eggs are inoculated on the 9th day of development and embryos are collected after 72 hours of infection, on the 12th day of development. The choice of this period is based on the ideal relationship between viral titer and embryo mortality [6, 7, 10]. Fox and Laemmert observed the YF 17DD viral proliferation peak in *Gallus gallus* at 72 hours post-infection [7]. According to Penna [10], three days of infection is the optimum condition for vaccine production while four days is also acceptable. Based on morphological observations, we did not detect any substantial increase in infection between 72 and 96 hours, except for the nervous system, where higher concentrations of positive cells appeared after 96 hours of infection.

We performed a virus sequencing analysis in order to confirm that PCR and immunofluorescence detections were really consistent with the presence of the YF 17DD virus. However, since this approach was not directed towards a single organ, we cannot discard the possibility of different YF 17DD subpopulation production in specific organs. Some authors suggest that the YF 17DD vaccine is not composed of a cloned population but actually a mixture of a few subpopulations [21–23]. As a result, the late enhancement of the nervous system infection could be either due to the need for more time for the YF 17DD virus to be produced by the neurons, or by the later production of a specific minority subpopulation that preferentially spreads in the brain.

The wild yellow fever virus is predominantly viscerotropic in humans and other primates, while it is neurotropic in mice and rabbits [20]. Although uncommon, there are reports of neurotropic disease associated with the yellow fever vaccine, mostly in children or immunocompromised patients [2, 24, 25]. Curiously, during the process of attenuation of the Asibi strain, after successive passages in mouse brain, the virus could be produced by neurons. Other passages in chicken embryo tissues without nervous system were needed to reduce this characteristic in the YFV 17D strain [25]. Our data demonstrate that the YFV 17DD infects cells of the nervous system in the chicken brain, spinal cord and peripheral nerves, which denotes relative neurocompetence to produce the YF 17DD virus. However, in this model the infection of the nervous system could be facilitated by the immaturity of the blood-brain barrier during this development stage [26].

An event that draws attention to our findings was the absence of both histopathological changes and viral antigen in the liver of the studied animals. The liver is the most affected organ in wild yellow fever affecting humans, other primates and golden hamsters [20, 27]. Similarly, in vaccine-associated viscerotropic disease cases, the liver is also the most affected organ [2]. In intraperitoneally inoculated Rhesus monkeys, Kupffer cells are infected 24 hours after inoculation [20]. In a model closer to our experiment, Fox and Laemmert reported, by functional tests, that the YF 17D-204 strain infected chicken embryo livers, since 72 hpi, even though this organ presented lower virus titers than all other organs studied [7]. The apparent contradiction between our data and those from this work could be explained by the possible viremia in the liver blood circulation. Additionally, it is also possible that some histopathological changes in the liver take place at later infection intervals.

The formation of muscle bundles in chicken embryos occurs between 8 and 18 days of development [28]. Between 8 and 10 days the peak myotube formation and consequent myoblast number decrease is reached [28, 29]. In this period of development, we observed after 48 hours of infection (11th day of development) positive myoblasts in muscle tissue and then, after 72 hours of infection (12th day of development), fully infected muscle bundles. In some fields, there were infected myoblasts associated with muscle bundles, but after 96 hours we detected only infected muscle fibers. Apparently, the first cells to be infected are the myoblasts with mesenchymal shaped cells, which will later form infected muscle bundles. As we have described before [8], at 72 hours of infection, muscle tissue can represent the major site of viral replication in the embryo because of its occupation area and the number of infected cells. It is known that muscle cells can be infected by arboviruses, such as the Chikungunya [30], Mayaro [31] and Ross River [32] viruses, leading to human clinical myalgia as a common element. The Chikungunya virus specifically infects human satellite cells [30], which are progenitor cells of muscles [33]. The importance of mesoderm-derived mesenchymal cells is also emphasized with regard to the YF 17DD viral chicken embryo infection, where there is a similar pattern of viral detection in lung parenchyma, and muscular wall cells of the gizzard, and yolk stalk. In addition, fibroblastoid cells of the dermis, perichondrium and meninges, also infected by the YFV 17DD, possess a mesenchymal origin as well. Our data suggest that these immature mesenchymal cells play an important role in the proliferation of the YFV 17DD. However, cells of other origins, such as kidney epithelial cells and neurons, can also be infected by the YFV 17DD.

Finally, we conclude that different tissues are involved in the course of the YFV 17DD chicken embryo infection for vaccine production. The muscle tissue is the first to demonstrate infection, and immature myoblast cells with a mesenchymal shape are noteworthy in early infection. Moreover, we observed an increase in infected nervous tissue at 96 hours, supporting vaccine production at 72 hours instead. We believe the data of this study elucidate important aspects of the pathology caused by the YF 17DD virus in chicken embryos, which may be helpful in the understanding and design of new strategies for vaccine production.
